# Evaluation of monitor unit calculation based on measurement and calculation with a simplified Monte Carlo method for passive beam delivery system in proton beam therapy

**DOI:** 10.1120/jacmp.v16i5.5419

**Published:** 2015-09-08

**Authors:** Kenji Hotta, Ryosuke Kohno, Kohsuke Nagafuchi, Hidenori Yamaguchi, Ryohei Tansho, Yoshihisa Takada, Tetsuo Akimoto

**Affiliations:** ^1^ Particle Therapy Division Research Center for Innovative Oncology, National Cancer Center Hospital East Kashiwa Chiba Japan; ^2^ Graduate School of Pure and Applied Sciences University of Tsukuba Tsukuba Japan

**Keywords:** passive proton therapy, dose per monitor unit

## Abstract

Calibrating the dose per monitor unit (DMU) for individual patients is important to deliver the prescribed dose in radiation therapy. We have developed a DMU calculation method combining measurement data and calculation with a simplified Monte Carlo method for the double scattering system in proton beam therapy at the National Cancer Center Hospital East in Japan. The DMU calculation method determines the clinical DMU by the multiplication of three factors: a beam spreading device factor $F_{\text{BSD}}$, a patient‐specific device factor $F_{\text{PSD}}$, and a field‐size correction factor $F_{\text{FS}(A)}$. We compared the calculated and the measured DMU for 75 dose fields in clinical cases. The calculated DMUs were in agreement with measurements in ±1.1% for all of 25 fields in prostate cancer cases, and in ±3% for 94% of 50 fields in head and neck (H&N) and lung cancer cases, including irregular shape fields and small fields. Although the $F_{\text{BSD}}$ in the DMU calculations is dominant as expected, we found that the patient‐specific device factor and field‐size correction also contribute significantly to the calculated DMU. This DMU calculation method will be able to substitute the conventional DMU measurement for the majority of clinical cases with a reasonable calculation time required for clinical use.

PACS number: 87.55.kh

## I. INTRODUCTION

Calibrating the dose per monitor unit (DMU) for individual patients is important to deliver the prescribed dose in radiation therapy. The DMU is defined as a ratio of irradiated dose at the isocenter to an amount of charge in an ionization reference chamber expressed in monitor units. This DMU for proton beam therapy depends on patient‐specific beam conditions, such as beam energy, the width of spread‐out Bragg peak (SOBP), the thickness of range shifter (RS), the shapes of a range compensator, and an aperture collimator. For patient‐specific beam conditions, most proton therapy facilities have been obtaining the DMU not by calculation in the treatment planning system, but by measurement in a water‐equivalent phantom representing the treatment condition. This is because it is difficult to accurately simulate the behavior of protons passing through complex combination of beam delivery devices and entering into the patient with possible lateral heterogeneity.^(1)^ Additionally, it takes much time and labor at present to measure the DMU for patient‐specific proton fields in clinical routine.

For DMU calculations, Kooy et al.^2^, ^3^ developed a semi‐empirical calculation method for a range modulated proton beam. It is based on the fact that the DMU depends on the ratio of the entrance dose and the reference dose. They obtained a result that the DMU depends on a single factor (R ‐ M) / M, where R is the distal range and M is modulation width. On the other hand, Sahoo et al.^(4)^ calculated the DMU based on measurements of dose at isocenter in various conditions of beam delivery devices. They expressed the DMU as a product of eight factors that are based on measurements for sampled conditions. The calculation accuracy of their method was within 2% for 99% of 623 distinct fields. Their methods calculate the DMU in homogeneous water‐equivalent phantoms, not in heterogeneous media such as a patient.

Currently, approaches with Monte Carlo simulations have been studied to improve accuracy of proton dose prediction in tissue heterogeneity.^5^, ^6^, ^7^, ^8^, ^9^, ^10^ From these studies, it was found that the DMU calculation method should consider the effect of heterogeneous media such as a patient and a range compensator in proton therapy in the same way heterogeneous correction is required for DMU calculation in photon therapy.^(11)^ Thus, we expect that the DMU calculation with the Monte Carlo method has a potential of solving these problems. However, since it takes much time to calculate the DMU with the full Monte Carlo method, reduction of calculation time is required to apply the Monte Carlo method for calculating the DMU in a patient routinely. Meanwhile, we have already developed a simplified Monte Carlo (SMC) method, with which we can quickly calculate dose distributions accurately.^12^, ^13^

In this paper, looking ahead to calculate the DMU in heterogeneous media as a final goal, we have developed, at present, a new DMU calculation method in a uniform phantom with the SMC method for the double scattering system of National Cancer Center Hospital East (NCCHE). We show results of comparison between the calculated and the measured DMUs in a water‐equivalent phantom for 75 clinical cases.

## II. MATERIALS AND METHODS

### A. Beam line

**Figure 1:**
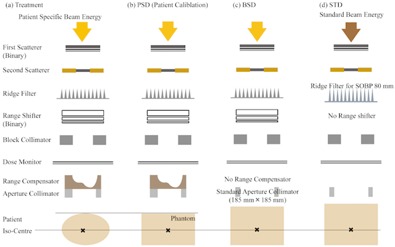
Scheme of beam line arrangement.

We measured and calculated proton dose distributions formed by the double scattering system of NCCHE.^(14)^ We used a proton beam with one of the energies 235, 190 or 150 MeV provided by a 235 MeV proton cyclotron and an energy selection system comprised of carbon degraders and a beam collimator. A treatment gantry with the double‐scattering system has beam‐shaping devices, as shown in Fig. 1. Thickness of the binary first scatterer and a type of second scatterer are determined by the proton energy. The maximum field size formed by the double‐scattering system is $200\times 200\;{\text{mm}}^{2}$. A ridge filter is used to form a spread‐out Bragg peak (SOBP). The SOBP widths can be selected from 30 mm to 100 mm at intervals of 10 mm. A transmission dose monitor is placed downstream of the ridge filter and binary range shifter. A patient aperture collimator is placed downstream of a range compensator to sharpen the lateral dose falloff in the peripheral region of PTV.

### B. Detectors

While a PTW 30013 was used for absolute dose measurement, a PTW 2D Array seven29 (PTW Freiburg GmbH, Freiburg, Germany) was used for relative dose measurements. The PTW 30013 is a Farmer type chamber with a sensitive volume of ϕ 6.1 mm×23.4 mm(0.6cc). It was used to measure the dose in a uniform water‐equivalent phantom under the uniform field conditions where patient‐specific devices (a range compensator and an aperture) were removed. For the patient‐specific QA, we measure the dose at the isocenter in a uniform water‐equivalent phantom under the nonuniform field condition with a patient aperture collimator and a range compensator. While the PTW 30013 was used for the field size larger than $4\times 4\;{\text{cm}}^{2}$, the PTW 31015 was used for the field size smaller than $4\times 4\;{\text{cm}}^{2}$. In this paper, data for fields more than $4\times 4\;{\text{cm}}^{2}$ were selected. The PTW 2D Array seven29 has 729 ionization chambers in a 10 mm pitch 27×27 array. Spezi et al.^(15)^ reported the successful application of this detector to radiation therapy and verified the performance. The sensitive volume of a unit chamber is 5 mm×5 mm×5 mm and the ionization chambers of the array are open to the air. The offset thickness from the entrance surface to the center of the sensitive volume was 8 mm in water‐equivalent length (WEL). The chamber array was used to measure the relative dose under both the uniform field condition and a possible nonuniform field condition that patient‐specific devices were inserted. To compare the calculations and measurements under the same conditions, we integrated the calculated dose distribution in a rectangular area with a detector cell size of 6.1 mm×23.4 mm for the PTW 30013 or 5 mm×5 mm for the PTW 2D Array seven29.

### C. Calculation of DMU

In NCCHE, the DMU measurement in a patient‐specific condition is made with a patient‐specific aperture collimator and a range compensator, as shown in Fig. 1(b). To derive the DMU in the patient‐specific conditions by calculation, we defined the DMU in the patient‐specific conditions (${\text{DMU}}_{\text{calc},\text{clinical}}$) as a product of the standard DMU (${\text{DMU}}_{\text{meas},\text{STD}}$) and a clinical beam delivering condition factor ($F_{\text{calc},\text{clinical}}$). The ${\text{DMU}}_{\text{meas},\text{STD}}$ is a DMU measured in our standard beam measurement (STD) conditions, as shown in Fig. 1(d). Details of the STD conditions are described in the Table 1. The $F_{\text{calc},\text{clinical}}$ can be written by multiplication of three factors: a beam‐spreading device factor ($F_{\text{BSD}}$), a patient‐specific device factor ($F_{\text{PSD}}$), and a field‐size correction factor ($F_{\text{FS}(A)}$) in the following way.

(1) $$DMU_{calc,clinical}\equiv DMU_{meas,STD}\times F_{calc,clinical}$$

(2) $$F_{calc,clinical}\equiv F_{BSD}\times F_{PSD}\times F_{FS(A)}$$

(3) $$F_{BSD}\equiv DMU_{meas,BSD}/DMU_{meas,STD}$$

(4) $$F_{PSD}\equiv DMU_{calc,PSD}/DMU_{calc,BSD}$$

(5) $$F_{FS(A)}\equiv f_{meas,FS(A)}/f_{calc,FS(A)}$$

(6) $$f_{calc,FS(A)}\equiv DMU_{calc,FS(A)}/DMU_{calc,FS(A=185)}$$

(7) $$f_{meas,FS(A)}\equiv DMU_{meas,FS(A)}/DMU_{meas,FS(A=185)}$$

The subscripts *STD, BSD, PSD*, and *FS* in equations denote beam delivery and measurement conditions, respectively, as summarized in Table 1; the prefixes, *meas* and *calc*, of the subscripts in the equations denote that the value was obtained by either measurement or calculation.

**Table 1:** Beam delivery and measurement conditions for STD, BSD, PSD, FS(A)

	*STD*	*BSD*	*PSD*	*FS(A)*
Energy	190 MeV	Patient‐specific	Patient‐specific	Patient‐specific
RS Thickness	0 mm	Patient‐specific	Patient‐specific	0 mm
SOBP Width	80 mm	Patient‐specific	Patient‐specific	80 mm
Collimator to Axis Distance	300 mm	300 mm	Patient‐specific	300 mm
Range Compensator	Nonexistent	Nonexistent	Patient‐specific	Nonexistent
Patient Collimator	$185\times 185\;{\text{mm}}^{2}$	$185\times 185\;{\text{mm}}^{2}$	Patient‐specific	$A\times A\;{\text{mm}}^{2}$
Target	Phantom	Phantom	Phantom	Phantom
Measurement Point	Center of SOBP	Center of SOBP	Patient‐ specific	Center of SOBP

The $F_{\text{BSD}}$ represents the effect of commonly used devices, such as the first and second scatterers, a range shifter, and a SOBP filter, on the DMU. It is defined as a ratio of measured DMUs in the BSD condition, as shown in Fig. 1(c) to that in the STD condition. In the BSD condition, while the beam spreading devices are set in the same way as in the patient‐specific condition, a patient‐specific aperture collimator is replaced by a standard collimator (a collimator with a square aperture of $185\times 185\;{\text{mm}}^{2}$), and a range compensator is removed. The $F_{\text{BSD}}$ is obtained by measurements since the combination of parameters of commonly used devices are limited.

The $F_{\text{PSD}}$ represents the effects of custom‐made devices for an individual patient (a patient‐specific aperture collimator and a range compensator) and an air‐gap between the collimator and the patient surface on the DMU. It is defined as a ratio of calculated DMUs by using the SMC in the BSD condition to that in the PSD condition, as shown in Fig. 1(b). In the PSD condition, all devices are set at a patient‐specific condition.

The $F_{\text{FS}(A)}$ corrects the discrepancy of $F_{\text{PSD}}$ for the smaller field size. The effect is called a field‐size effect, as mentioned in the Materials & Methods section C.3 below. This factor is defined as a ratio of the measured field‐size factor $f_{\text{meas},\text{FS}(A)}$ to the calculated field‐size factor $f_{\text{calc},\text{FS}(A)}$ expressed as a function of the mean of distances between edge points of an aperture collimator and the isocenter. The $f_{\text{meas},\text{FS}(A)}$ (or $f_{\text{calc},\text{FS}(A)}$) is defined as a ratio of measured (or calculated) DMUs in the FS condition with square‐shaped aperture of “A” mm a side to that with square‐shaped aperture of 185 mm a side.

#### C.1 Measurement of beam spreading device factor: $F_{\text{BSD}}$

The beam spreading device factor $F_{\text{BSD}}$ is a ratio of the ${\text{DMU}}_{\text{meas},\text{BSD}}$ to ${\text{DMU}}_{\text{meas},\text{STD}}$. We measured the ${\text{DMU}}_{\text{meas},\text{BSD}}$ in a polyethylene phantom at the position corresponding to the center of SOBP identical to the isocenter using the PTW 30013. The thickness of the phantom on the PTW 30013 was calculated by an equation: proton range ‐SOBP/2 ‐ RS)/WELR, where the SOBP and RS are the water‐equivalent width of spread‐out Bragg peak and the water‐equivalent thickness of the range shifter, respectively. The WELR is the water‐equivalent thickness ratio of the polyethylene phantom.

**Figure 2:**
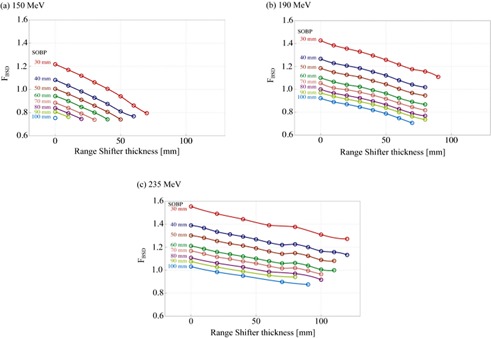
Change of $F_{\text{BSD}}$ for three energies.

We measured the ${\text{DMU}}_{\text{meas},\text{BSD}}$ for eight ridge filters with the SOBP width from 30 mm WEL to 100 mm WEL at intervals of 10 mm WEL for three energies. For each proton energy, the RS thickness was varied from 0 mm WEL to the maximum value up to 120 mm WEL at intervals of 10 mm or 20 mm WEL, depending on the width of the SOBP. Figure 2 shows the measurement results of the FBSD. All datasets were normalized by the dose at the isocenter in the STD condition. The $F_{\text{BSD}}$ decreased with an increase in the RS thickness and an increase in the SOBP width due to the following three reasons. Firstly, since the lower energy protons passing through the thicker RS deposit larger energy in the ionization reference chamber, the number of protons reaching the center of SOBP decreases for the same MU, resulting in decrease in $F_{\text{BSD}}$. Secondly, a ridge filter with a larger SOBP width increases the average energy of protons reaching the isocenter, resulting in a decreased dose at the isocenter for the same MU. In addition, since average energy of protons entering into the ionization reference chamber decreases with an increase in the SOBP width, the number of protons reaching the center of SOBP decreases for the same MU. Thirdly, as the RS thickness and the SOBP width increases, more material is inserted in the beam course. Then proton scattering and nuclear interaction increase in the inserted material, resulting in decrease of proton fluence at the isocenter.

#### C.2 Calculation of patient‐specific device factor: F_PSD_

For DMU_calc,PSD_ and DMU_calc,BSD_ calculations, we used the simplified Monte Carlo method (SMC).^12^, ^13^ It tracks individual protons in a range compensator, an aperture collimator, and a phantom by calculating the range loss and scattering in materials using the water‐equivalent model^(16)^ and the Highlands's equation.^(17^) Scattering effect in commonly used devices is integrated and expressed by using the single Gaussian effective‐source model with the model parameters determined by measurements.^(18,19^) The measured depth‐dose distribution in water was used to obtain the relative dose deposit in a patient voxel. The voxel dimension in the phantom was taken as 2 mm×2 mm×2 mm and the number of particles is determined so that the statistical error was less than 1%. To shorten the calculation time, we have adopted general‐purpose computing on graphics processing units (GPGPU) technique in this DMU calculation.^(20^) Since the calculation time is about 1 min for each proton field, this method is practical in clinical use.

#### C.3 Measurement of field‐size factor: $F_{\text{FS}(A)}$

The field‐size factor $F_{\text{FS}(A)}$ is the ratio of $f_{\text{meas},\text{FS}(A)}$ to $f_{\text{calc},\text{FS}(A)}$. We measured the ${\text{DMU}}_{\text{meas},\text{FS}(A)}$ at the isocenter in the FS setting for each of three energies, using a collimator with a square‐shaped aperture. We prepared five collimators with an aperture side of 40, 50, 70, 100, 185 mm and measured the ${\text{DMU}}_{\text{meas},\text{FS}(A)}$ for each of them. We also calculated the dose distributions under the same conditions. We used the SMC as stated above for calculations. The calculation voxel dimension in the phantom was taken as 2 mm×2 mm×2 mm. The number of particles is determined so that the statistical error is less than 1%.

**Figure 3:**
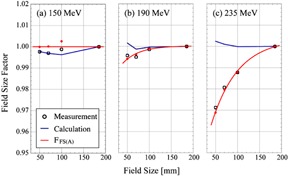
Comparison of calculation and measurement results for field‐size effect. The circles show the measurement results, the blue solid line shows the calculation results, and the red thick line shows the F_(FS(A)_) that is the ratio of measurement and calculation.

Figure 3 shows the $F_{\text{FS}(A)},f_{\text{meas},\text{FS}(A)},f_{\text{calc},\text{FS}(A)}$. Datasets for each of three energies were normalized to unity for the case where a collimator with an aperture side of 185 mm was used. The circles show the measurement results. The blue solid line shows the calculation results using the SMC. The red thick line shows the $F_{\text{FS}(A)}$ is the ratio of $f_{\text{meas},\text{FS}(A)}$ to $f_{\text{calc},\text{FS}(A)}$. The $f_{\text{meas},\text{FS}(A)}$ is the measured DMU in the FS(A) condition normalized by the measured DMU in the FS(185) condition. The $f_{\text{calc},\text{FS}(A)}$ is the calculated one corresponding to the $f_{\text{meas},\text{FS}(A)}$. The measurement results showed that the ${\text{DMU}}_{\text{meas},\text{FS}(A)}$ decreased with a decrease in the aperture size of the collimator for proton energies of 190 and 235 MeV. The ${\text{DMU}}_{\text{meas},\text{FS}(A)}$ decreases by about 5% when the aperture size of collimator varies from 185 mm to 40 mm for 235 MeV protons. While the measured aperture effect was less than 1% for protons with energies, 190 MeV and 150 MeV, it was significant for 235 MeV protons. On the other hand, the calculated ${\text{DMU}}_{\text{calc},\text{FS}(A)}$ was almost 1.0 for the collimator aperture size larger than 40 mm and began to decrease for the collimator aperture size less than 30 mm.

In general, the dose at the isocenter consists of on‐axis contribution from protons entering in the phantom along the beam central axis, and off‐axis contribution from protons entering in the phantom at neighboring positions of the beam central axis and reaching the isocenter due to the initial angular spread and scattering in the phantom. In larger field sizes, the protons that enter in the phantom at the beam central axis and escape from the axis at the isocenter balance the off‐axis contribution. In such cases, no field‐size effect is observed. As the field size shrinks, the balance is broken due to decreased off‐axis contribution and the field‐size effect begins to appear. On the other hand, edge‐scattered protons in the collimator begin to contribute to the dose at the isocenter as the distance between the beam central axis and the collimator aperture edge gets smaller. Thus the field‐size effect is a net result of such complex phenomena. It is apparent that the observed discrepancy between the measured and calculated field‐size effect implies that the current SMC calculation model is not enough to reproduce the field‐size effect. Although we consider at present that the possible causes of the discrepancy may be the current Gaussian model of initial angular distribution of protons and/or the model of the edge scattering in the collimator, it is an open problem for future study. At present, we correct the difference by the field‐size factor, $F_{\text{FS}(A)}$. For an irregular‐shaped aperture found in the clinical case, a mean aperture radius is calculated and the field‐size factor $F_{\text{FS}(A)}$ for a collimator with the same mean aperture radius is used for the correction. We defined the mean aperture radius as a mean of distances between all the collimator edge points and the isocenter. The collimator edge points were given in the same resolution of the planning CT image.

### D. DMU measurement in clinical conditions

We compared the calculated and measured dose distribution on the isocenter plane for seven clinical fields of H&N, prostate, and lung cancer cases. We mounted a stack of polyethylene phantoms on the PTW 2D array seven29 detector to measure a dose distribution at the same water‐equivalent depth as the water‐equivalent depth of the isocenter for clinical field. The beam line device parameters and the phantom thickness are shown in Table 2, corresponding to the seven clinical cases.

We also compared the measured and calculated results for 75 clinical fields of H&N, lung, and prostate cancer cases (25 fields for each), including the aforementioned seven fields. We redefined $F_{\text{meas},\text{clinical}}$ and $F_{\text{calc},\text{clinical}}$ by the following equations:

(8) $$F_{meas,clinical}\equiv DMU_{meas,clinical}/DMU_{meas,STD}$$

(9) $$F_{calc,clinical}\equiv F_{BSD}\times F_{PSD}\times F_{FS(A)}$$

Here, $DMU_{meas,clinical}$ is a DMU measured in a patient‐specific beam condition with a patient‐aperture collimator and a range compensator. The dose was measured in a polyethylene phantom at the water‐equivalent depth of isocenter by using the PTW 30013.

**Table 2:** Beam delivery and measurement conditions for individual clinical fields

	*Energy (MeV)*	*SOBP Width (mm)*	*RS Thickness (mm‐WEL)*	*PE Thickness (mm)*
Prostate 1	235	60	55.1	162
Prostate 2	235	60	48.7	169
Lung 1	190	50	62.6	66
Lung 2	150	50	21.5	52
H&N 1	150	70	22.0	22
H&N 2	150	70	26.1	31
H&N 3	150	60	8.7	20

## III. RESULTS & DISCUSSION

**Figure 4:**
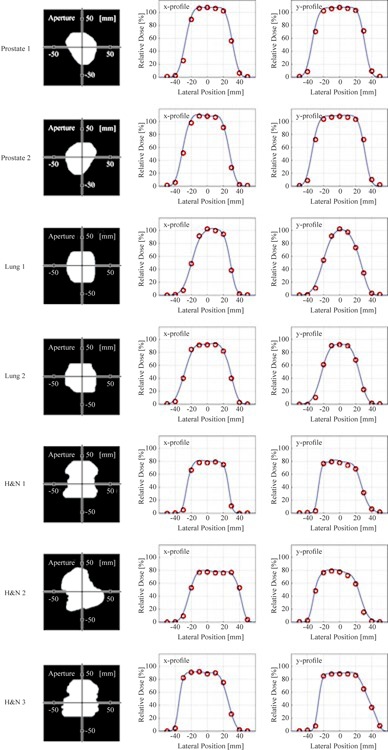
Measured and calculated relative dose distributions for 7 clinical beams. Left figures show the shape of aperture collimators. Center and right figures show x and y dose profiles, respectively. Red circles are measured doses and blue lines are calculated dose distributions.

Figure 4 shows the measured and calculated relative dose distributions for the seven clinical fields. All datasets were normalized by the ${\text{DMU}}_{\text{meas},\text{STD}}$. Measured dose distributions were in good agreement with calculated ones. Some of them, such as the X profile of H&N 3 and the Y profile of lung 1, had nonuniform dose distributions around the isocenter. In these cases, the measured DMU may have included a dose error originating from misalignment of the dose monitor relative to the range compensator in the nonuniform dose region. In order to reduce such an error, the dose monitor should be placed in a more uniform dose region.

**Figure 5:**
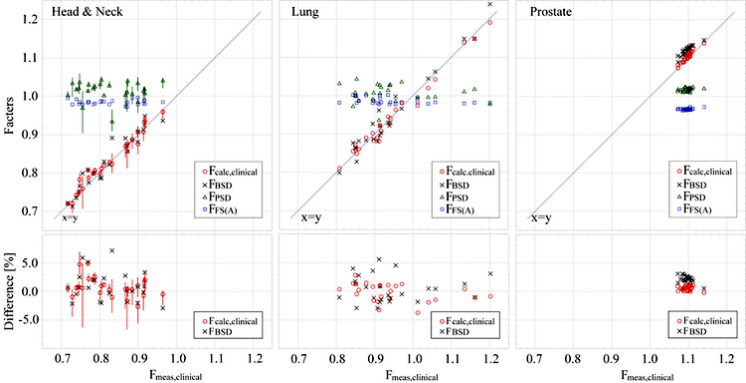
(upper) The relation between measured ($F_{\text{meas},\text{clinical}}$) and calculated factors ($F_{\text{calc},\text{clinical}},F_{\text{BSD}},F_{\text{PSD}}$, F_FS_) of 75 proton dose fields. (lower) The difference of $F_{\text{calc},\text{clinical}}$ or $F_{\text{BSD}}$ from $F_{\text{meas},\text{clinical}}$. The error bars for $F_{\text{PSD}}$ and $F_{\text{calc},\text{clinical}}$ were calculated from peak‐to‐peak dose variation within a cubic region of 2 mm on a side around the measurement point.

Figure 5 shows the relation between the $F_{\text{meas},\text{clinical}}$ and other calculated or measured factors ($F_{\text{calc},\text{clinical}},F_{\text{BSD}},F_{\text{PSD}},F_{\text{FS}(A)}$) of 25 proton dose fields for each of three clinical sites. Upper graphs show the values of $F_{\text{calc},\text{clinical}},F_{\text{BSD}},F_{\text{PSD}}$, and $F_{\text{FS}(A)}$ versus $F_{\text{meas},\text{clinical}}$. The error bars for the $F_{\text{PSD}}$ and $F_{\text{calc},\text{clinical}}$ were calculated from peak‐to‐peak dose variation within a cubic region of 2 mm on a side around the isocenter obtained by the calculated dose distribution.

Since the statistical errors were estimated to be smaller than 0.1% for the detection volume of the Farmer chamber, we ignored them. We notice from the graphs in the Fig. 5 that the $F_{\text{BSD}}$ is a dominant factor of the $F_{\text{calc},\text{clinical}}$, since the $F_{\text{PSD}}$ and $F_{\text{FS}(A)}$ were almost 1.0. Here, we focus on the $F_{\text{calc},\text{clinical}}$ and $F_{\text{BSD}}$ to evaluate contribution of each factor in the calculation. Lower graphs of Fig. 5 show the differences of the $F_{\text{calc},\text{clinical}}$ and $F_{\text{BSD}}$ from the $F_{\text{meas},\text{clinical}}$. The mean, standard deviation (SD), maximum, and minimum of the difference are summarized in Table 3 for the $F_{\text{PSD}}$ and $F_{\text{FS}(A)}$. These results show that agreement between the $F_{\text{calc},\text{clinical}}$ and $F_{\text{meas},\text{clinical}}$ is better than that between the $F_{\text{BSD}}$ and $F_{\text{meas},\text{clinical}}$. Thus the $F_{\text{PSD}}$ and $F_{\text{FS}(A)}$ serve to improve calculation accuracy of the DMU significantly.

For prostate cancer cases, field sizes clustered around 5 cm. The $F_{\text{calc},\text{clinical}}$ agrees with $F_{\text{meas},\text{clinical}}$ within ±1.1% of the $F_{\text{meas},\text{clinical}}$ for all fields. Values of the $F_{\text{meas},\text{clinical}}$ cluster around 1.1 since the anatomic location and shape of the target are very similar patient by patient. The mean value of $F_{\text{BSD}}$ minus $F_{\text{meas},\text{clinical}}$ is systematically larger than that of the $F_{\text{calc},\text{clinical}}$ minus $F_{\text{meas},\text{clinical}}$, and the both SDs are very small. Therefore, in prostate cancer cases, we could obtain the $F_{\text{calc},\text{clinical}}$ only by adding the difference of the mean values as a correction factor to the $F_{\text{BSD}}$. We also notice in the rightmost upper graph of Fig. 5 that the difference comes mainly from the significant deviation of the $F_{\text{FS}(A)}$ from unity. It corresponds to the fact that the $F_{\text{FS}(A)}$ deviates from unity mostly in the highest energy, 235 MeV, used for prostate cancers.

**Table 3:** Mean, SD, maximum (each of over‐ and underestimation) of difference of calculation from measurement for 75 clinical dose fields

	*No. of Fields*		*Mean*	*SD*	*Max*.	*Min*.
H&N	25	$F_{\text{calc},\text{clinical}}$ ‐ $F_{\text{meas},\text{clinical}}$	0.78%	1.73%	4.93%	−2.70%
		*F*_BSD_ ‐ $F_{\text{meas},\text{clinical}}$	0.92%	2.58%	7.09%	−2.91%
Lung	25	$F_{\text{calc},\text{clinical}}$ ‐ $F_{\text{meas},\text{clinical}}$	−0.19%	1.58%	2.79%	−3.76%
		*F*_BSD_ ‐ $F_{\text{meas},\text{clinical}}$	0.47%	2.37%	5.56%	−2.93%
Prostate	25	$F_{\text{calc},\text{clinical}}$ ‐ $F_{\text{meas},\text{clinical}}$	0.53%	0.42%	1.26%	−0.21%
		*F*_BSD_ ‐ $F_{\text{meas},\text{clinical}}$	2.21%	0.55%	3.08%	0.54%

**Figure 6:**
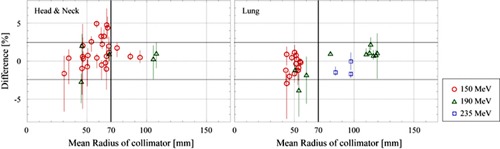
The dependence of difference between $F_{\text{meas},\text{clinical}}$ and $F_{\text{calc},\text{clinical}}$ on field size. The error bars were calculated from peak‐to‐peak dose variation within a cubic region of 2 mm on a side around the measurement point.

For the lung and H&N cancer cases, the field sizes distribute between 3 cm to 12 cm in mean radius, as shown in Fig. 6. The $F_{\text{calc},\text{clinical}}$ agrees with $F_{\text{meas},\text{clinical}}$ within ±2.2% of the $F_{\text{meas},\text{clinical}}$ for 94% of 50 fields. The variation of the difference, $F_{\text{calc},\text{clinical}}$ minus $F_{\text{meas},\text{clinical}}$, is larger than that of prostate cancer cases, as shown in Table 3. For H&N and lung cancer cases, the SDs were 1.7% and 1.6%, and maximum differences in plus and minus sides were +4.9%,−2.7% and +2.8%,−3.8%, respectively.

We examined the relation between effect of the possible setup error on the error of the difference and the aperture size of the collimator. Figure 6 shows dependence of the difference and its error on the mean aperture radius of collimator for each of the three proton energies. Errors larger than 2.5% are observed for the collimators with a mean aperture radius less than 70 mm. In these cases, measurement uncertainties caused by the setup error in the region with a nonuniform dose distribution are as large as those shown by the error bars. We also consider here the possible inaccuracy of the present model of the field‐size correction. In the present model, we assume that the $F_{\text{FS}(A)}$ is a function of two parameters: the initial proton energy and field size. However, it may also be affected by other conditions: the RS thickness, SOBP width, and the depth of isocenter. Since the error of difference is large for the collimator with small aperture size, as shown in Fig. 6, and the field‐size effect is significant for collimator with smaller aperture size, as shown in Fig. 3, more study for the small proton field will be required to improve calculation accuracy of the DMU.

## IV. CONCLUSIONS

We have developed a DMU calculation method combining measurement data and calculation with a simplified Monte Carlo method for the double scattering system of NCCHE. The ${\text{DMU}}_{\text{clinical}}$ is represented by the ${\text{DMU}}_{\text{meas},\text{STD}}$ multiplied by F_clinical_. The $F_{\text{clinical}}$ is defined by multiplication of three factors: the beam spreading device factor $F_{\text{BSD}}$, the patient‐specific device factor $F_{\text{PSD}}$, and the field‐size factor $F_{\text{FS}(A)}$. The $F_{\text{BSD}}$ is obtained by measurements for limited combinations of three beam delivery conditions: the proton energy, the SOBP width, and the RS thickness. The $F_{\text{PSD}}$ is obtained by calculations using the SMC method. The $F_{\text{FS}(A)}$ is obtained empirically, based on the measurements. Although the $F_{\text{BSD}}$ is a dominant factor in the DMU calculation method, the $F_{\text{PSD}}$ and the $F_{\text{FS}(A)}$ also contribute significantly to the DMU calculation. The calculated DMUs agreed with measurements within ±1.1% for all of 25 fields for prostate cancer cases and within ±3% for 94% of 50 fields for H&N and lung cancer cases. Since the calculation time is within 1 min for each field, this method will be applicable to routine clinical use. Therefore, this method can be applied safely to determination of the DMU for all prostate cancer cases. In addition, it can be applied to determination of the DMU for most of the H&N and lung cancer cases.

## ACKNOWLEDGMENTS

We are grateful to Dr. Yuka Matsuzaki, Dr. Shie Nishioka, M.Sc. Yuya Sugama, and Dr. Tomoko Miyagishi for their useful discussion and technical support. The authors would also like to thank Hiroyuki Suzuki, Atsushi Sakamoto, Tetsurou Kawaguchi, Keigo Sadano, Hideki Watanabe, and Masaki Shinoda, SHI Accelerator Service Ltd., for experimental support. This work was supported by JSPS Grant‐in‐Aid for Scientific Research (C) Grant Number 26460739. This study was also supported in part by the National Cancer Center Research and Development Fund (25‐A‐10) and Health Science Research Grants from the Ministry of Health and Welfare.
